# Stereotactic body radiotherapy using gated radiotherapy with real-time tumor-tracking for stage I non-small cell lung cancer

**DOI:** 10.1186/1748-717X-8-69

**Published:** 2013-03-21

**Authors:** Tetsuya Inoue, Norio Katoh, Rikiya Onimaru, Shinichi Shimizu, Kazuhiko Tsuchiya, Ryusuke Suzuki, Jun Sakakibara-Konishi, Naofumi Shinagawa, Satoshi Oizumi, Hiroki Shirato

**Affiliations:** 1 Department of Radiation Medicine, Hokkaido University Graduate School of Medicine, North 15 West 7, Kita-ku, Sapporo 060-8638, Japan; 2 Department of Medical Physics, Hokkaido University Graduate School of Medicine, Sapporo, Japan; 3 Department of Respiratory Medicine, Hokkaido University Graduate School of Medicine, Sapporo, Japan

**Keywords:** Stereotactic body radiotherapy, Radiation pneumonitis, Non-small cell lung cancer, Real-time tumor-tracking, Tumor motion, Gated radiotherapy

## Abstract

**Background:**

To clarify the clinical outcomes of two dose schedule of stereotactic body radiotherapy (SBRT) for stage I non-small cell lung cancer (NSCLC) using a real-time tumor-tracking radiation therapy (RTRT) system in single institution.

**Methods:**

Using a superposition algorithm, we administered 48 Gy in 4 fractions at the isocenter in 2005–2006 and 40 Gy in 4 fractions to the 95% volume of PTV in 2007–2010 with a treatment period of 4 to 7 days. Target volume margins were fixed irrespective of the tumor amplitude.

**Results:**

In total, 109 patients (79 T1N0M0 and 30 T2N0M0). With a median follow-up period of 25 months (range, 4 to 72 months), the 5-year local control rate (LC) was 78% and the 5-year overall survival rate (OS) was 64%. Grade 2, 3, 4, and 5 radiation pneumonitis (RP) was experienced by 15 (13.8%), 3 (2.8%), 0, and 0 patients, respectively. The mean lung dose (MLD) and the volume of lung receiving 20 Gy (V20) were significantly higher in patients with RP Grade 2/3 than in those with RP Grade 0/1 (MLD p = 0.002, V20 p = 0.003). There was no correlation between larger maximum amplitude of marker movement and larger PTV (r = 0.137), MLD (r = 0.046), or V20 (r = 0.158).

**Conclusions:**

SBRT using the RTRT system achieved LC and OS comparable to other SBRT studies with very low incidence of RP, which was consistent with the small MLD and V20 irrespective of tumor amplitude. For stage I NSCLC, SBRT using RTRT was suggested to be reliable and effective, especially for patients with large amplitude of tumor movement.

## Background

Stereotactic body radiotherapy (SBRT) with high local dose has been applied to extra-cranial diseases such as peripheral stage I non-small cell lung cancer (NSCLC) and has been reported to provide excellent local control and survival compatible with surgery [1,2].

Respiratory motion of lung tumors has been one of the largest uncertainties in the radiotherapy of lung cancers [3]. Considering respiratory motion, there are several irradiation methods: (i) irradiation with breath coaching and holding [4-7], (ii) irradiation with respiratory gating using external surrogates with four-dimensional CT [7], (iii) immobilization in the stereotactic body frame (Elekta, Stockholm, Sweden) with an abdominal compression device to limit respiratory motion during radiotherapy [8,9], (iv) irradiation using planning CT with a slow scan technique [10,11], (v) pursuing irradiation with some prediction models [12-17], (vi) and real-time tumor-tracking radiotherapy (RTRT) system [18].

A prototype RTRT was developed at our institute in 1999 to increase the precision of irradiation for moving lung tumors. Real-time tumor-tracking radiotherapy comprises two different concepts: (i) pursuing irradiation where the therapeutic beam changes its direction during treatment; and (ii) interrupting irradiation where the therapeutic beam does not change its direction during treatment. The prototype RTRT system used the interrupting irradiation method. We clinically used the prototype RTRT system in which the linear accelerator is gated to irradiate the tumor only when the implanted fiducial marker is within 2 mm from its planned position [19,20]. We have published a study showing steep dose–response curve between 40 Gy and 48 Gy in four fractions in one week in patients with stage I NSCLC treated with RTRT between 2000 and 2005 in our institution [21]. In that study, we showed that 48Gy at the isocenter with 5 mm CTV-PTV margin, irrespective of amplitude of tumor motion, was safe and effective. Since then, dose calculation software has been upgraded to superposition algorithm from Clarkson algorithm, resulting in a certain difference in dose prescription method. The aim of the present study was to clarify the clinical outcomes of SBRT for stage I NSCLC using this RTRT system using dose calculation method with inhomogeneity correction between 2005 and 2010.

## Methods

### Patient characteristics

There were 109 patients (79 T1N0M0 and 30 T2N0M0) who received SBRT for stage I NSCLC using RTRT system at our institution between June 2005 and November 2010. Diagnosis of stage I NSCLC was based on whole-body CT and brain MRI. Of 109 patients, Fluoro-deoxy-glucose (FDG)-positron emission tomography (PET) was performed in 71 patients (65%). One hundred three patients had biopsy-proven NSCLC. The other six patients were biopsy-unproven, but clinically diagnosed as NSCLC according to evidence of interval progression on at least two serial CT imaging studies and/or increased FDG uptake on PET scan. The patient characteristics are given in Table 1.

**Table 1:** Patient characteristics

**Characteristics**	**Value**
Age (years)	
Median	78
Range	47-90
Gender (n)	
Male	74
Female	35
Histology (n)	
Adenocarcinoma	65
Squamous cell carcinoma	29
Large cell carcinoma	1
Unclassified NSCLC	8
Histologically unproved	6
Tumor size (mm)	
Median	22
Range	7-65
Tumor site (n)	
Upper lobe	50
Middle lobe	13
Lower lobe	46
T stage (n)	
T1a	47
T1b	32
T2	30
FDG-PET before SBRT (n)	
yes	71
no	38
Dose prescription (n)	
48Gy/4fr at isocenter	30
40Gy/4fr to the 95% volume of PTV	79

Abbreviation: *NSCLC*, non-small cell lung cancer.

### SBRT technique

All patients received SBRT to NSCLC using the RTRT system. The RTRT system has been described in detail elsewhere [18-20]. In brief, 1.5-mm gold markers were implanted near the tumor by bronchoscopy. CT scans were taken with the patients holding their breath at the end of normal expiration. The gross tumor volume (GTV) was defined using multi-slice CT taken at the end of expiration during breath holding. Giraud et al. reported that CTV margins must be increased to 8 mm and 6 mm for adenocarcinoma and squamous cell carcinoma [22]. Based on their report, we defined the clinical target volume (CTV) as the GTV plus 6 mm for squamous cell carcinoma, 8 mm for adenocarcinoma, and 5 mm for others where no appropriate basic literature had existed. The planning target volume (PTV) was the CTV plus a 5-mm margin irrespective of the amplitude of the tumor. The PTV margins were fixed irrespective of the tumor amplitude because RTRT is gated to irradiate the tumor only when the implanted fiducial marker is within 2 mm from its planned position. Therefore, the target volume did not increase when the tumor movement was large, especially in the lower lobe. In our RTRT system, the irradiated volume depended only on tumor size. The position of multileaf collimator sets was usually the PTV plus a 5-mm margin.

Using a superposition algorithm, we administered 48 Gy in 4 fractions at the isocenter in 2005–2006 (n = 30) and 40 Gy in 4 fractions to the 95% volume of PTV in 2007–2010 (n = 79) with a treatment period of 4 to 7 days. Isocenter dose of 40 Gy in 4 fractions to the 95% volume of PTV was approximately ranged from 45 to 50 Gy.

### Follow-up after SBRT

Follow-up visits were usually every 3 months after SBRT. CT scans were usually performed every 3 to 6 months after SBRT. Distinguishing between residual tumor tissue and radiation fibrosis was difficult. Local disease recurrence was considered to have occurred only when enlargement of the local tumor continued for > 6 months on follow-up CT scans. FDG-PET and/or histologic confirmation was recommended when local recurrence was suspected, but this was not mandatory. Absence of local disease recurrence was defined as locally controlled disease.

### Ethical considerations

Written informed consent to receive SBRT was obtained from all patients. This retrospective study was approved by the ethics committee of Hokkaido university hospital. This study was performed in accordance with the 1975 Declaration of Helsinki, as revised in 2000.

### Statistical analysis

The overall survival (OS) and the local control (LC) rates were calculated using the Kaplan-Meier method. The log-rank test was used to calculate the statistical significance of differences. Multivariate analysis was performed using a Cox proportional hazards regression model. The difference of means was analyzed with Student’s t-test. A value of p < 0.05 was considered to be statistically significant. R version 2.14.2 with the survival packages (R project for statistical computing, Vienna, Austria) was used for statistical analyses.

## Results

### Local control and survival

**Figure 1:**
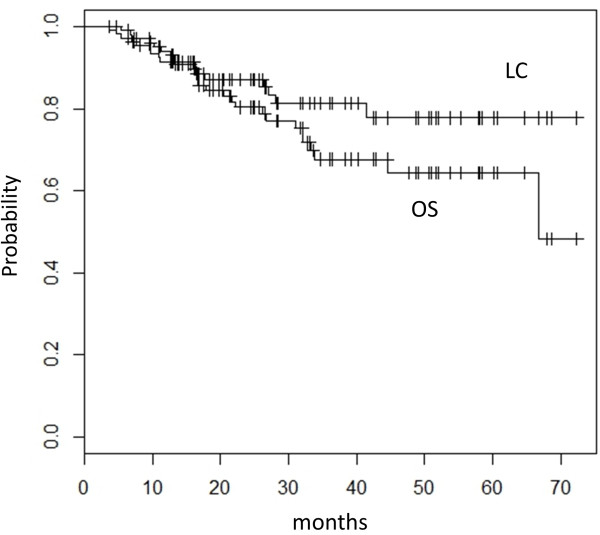
Kaplan-Meier actuarial overall survival (OS) and local control (LC) rate.

**Figure 2:**
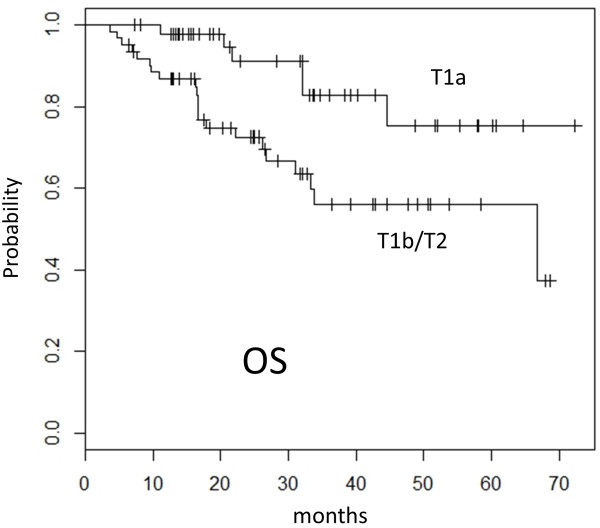
**Kaplan-Meier curve of overall survival (OS) rates for patients with T1a (n = 47) and T1b or T2 (n = 62). **Significant statistical difference was found (p = 0.01) between the two groups.

The TNM classification and clinical staging were determined according to the union of international cancer control (UICC) seventh edition. With a median follow-up period of 25 months (range, 4 to 72 months), the 3-year and 5-year local control rate (LC) was 81% (95% confidence interval (CI), 73-91%) and 78% (95% confidence interval (CI), 68-90%), respectively. The 3-year and 5-year overall survival rate (OS) was 68% (95% CI, 57-80%) and 64% (95% CI, 53-78%), respectively (Figure 1). In patients with T1a (tumor diameter ≤ 20 mm), the 3-year and 5-year LC was 92% (95% CI, 83-100%) and 83% (95% CI, 67-100%), and the 3-year and 5-year OS was 83% (95% CI, 70-98%) and 75% (95% CI, 58-97%). The OS in patients with T1a was significantly better compared with 56% (95% CI, 42-74%) for those with T1b or T2 (tumor diameter > 20 mm) (Figure 2). Univariate analysis showed gender and FDG-PET before SBRT to be prognostic factors in LC and tumor size in OS (Table 2). There was no statistical significant difference in OS between patients who underwent FDG-PET before SBRT and those who did not. No statistically significant difference was observed in OS between patients with pathological diagnosis and those without it. There was no statistically significant difference in LC and OS between patients treated with 48 Gy in 4 fractions at isocenter and those with 40 Gy in 4 fractions to the 95% volume of PTV. Multivariate analysis demonstrated that no variable remained as a prognostic factor for LC and only the tumor diameter ( ≤ / > 2 cm) was a significant prognostic factor for OS (p = 0.03) (Table 3).

**Table 2:** Univariate analyses for local control (LC) and overall survival (OS)

	**P value**	
**Variables**	**LC**	**OS**
Tumor site		
Lower lobe vs. others	0.25	0.16
Gender		
Female vs. others	0.02*	0.66
T stage		
T1a vs. others	0.06	0.01*
Primary histology		
Adenocarcinoma vs. others	0.16	0.53
Maximum amplitude of marker movement		
≥ 3 cm vs. < 3 cm	0.06	0.16
FDG-PET before SBRT (n)		
yes vs. no	0.03*	0.30
Dose prescription (n)		
48Gy/4fr at isocenter vs. 40Gy/4fr to the 95% volume of PTV	0.35	0.38

*, significant (p < 0.05).

**Table 3:** Multivariate analyses for local control (LC) and overall survival (OS)

	**P value**	
**Variables**	**LC**	**OS**
Gender		
Female vs. others	0.05	0.84
T stage		
T1a vs. others	0.25	0.03*
Maximum amplitude of the marker movement		
≥ 3 cm vs. < 3 cm	0.28	0.53

*, significant (p < 0.05).

### Toxicities

**Figure 3:**
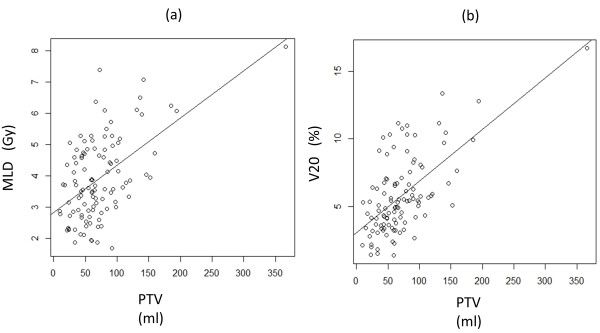
(a) Correlation with planning target volume (PTV) and mean lung dose (MLD), (b) Correlation with PTV and the volume of lung receiving 20 Gy (V20).

Adverse effects were graded according to the Common Toxicity Criteria for Adverse Events, version 3.0. Grade 2, 3, 4, and 5 radiation pneumonitis (RP) was experienced by 15 (13.8%), 3 (2.8%), 0, and 0 patients, respectively. The mean lung dose (MLD) (of lung volume minus PTV) was 4.0 ± 1.4 Gy in total. MLD was 4.8 ± 1.6 Gy in patients with RP Grade 2/3, compared with 3.8 ± 1.3 Gy in those with RP Grade 0/1. The volume of lung receiving 20 Gy (V20) (of lung volume minus PTV) was 5.8 ± 2.3% in total. V20 was 7.6 ± 3.3% in patients with RP Grade 2/3, compared with 5.4 ± 2.6% in those with RP Grade 0/1, respectively. MLD and V20 were significantly higher in patients with RP Grade 2/3 than in those with RP Grade 0/1 (MLD p = 0.002, V20 p = 0.003). There was a strong correlation between larger PTV and larger MLD (r = 0.535, p < 0.001) and V20 (r = 0.627, p < 0.001) (Figure 3). There was no significant difference between the patients with tumor at lower lobe and those with tumor at other lobes in the MLD, V20, and the incidence of Grade 2 or higher RP. Grade 2 intercostal neuralgia occurred in 6 patients (5.5%). Grade 5 infectious pneumonia occurred in one patient; he had experienced Grade 3 RP after SBRT for the T1b tumor at the upper lobe. RP was relieved by intravenous infusion of corticosteroids; however, his case was complicated by cytomegalovirus pneumonia, which caused his death. This infectious pneumonia may be produced due to immunosuppression caused by steroid therapy; therefore this might be a treatment-related death. No other adverse effects of Grade 2 or greater were observed.

### Insertion of the gold markers by bronchoscopy

No complications were occurred when gold markers were implanted near the tumor by bronchoscopy. No inter-fractional migrations of gold markers were occurred.

### Amplitude of respiratory tumor motion

**Figure 4:**
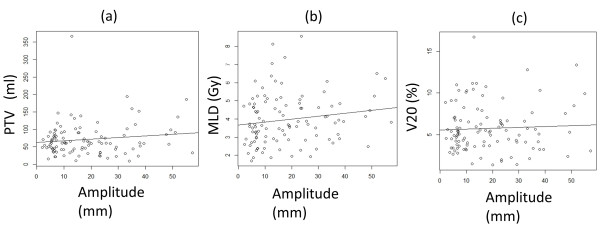
(a) Correlation with maximum amplitude of marker movement and planning target volume (PTV), (b) Correlation with maximum amplitude of marker movement and mean lung dose (MLD), (c) Correlation with maximum amplitude of marker movement and the volume of lung receiving 20 Gy (V20).

The mean of maximum amplitude of the marker movement of the lower lobe was 27.2 ± 13.8 mm, which was significantly greater than that of the upper lobe (11.3 ± 9.9 mm, p < 0.001). There was no significant difference between the lower lobe and other lobes in the 5-year LC and OS (Table 2). There was no correlation between larger maximum amplitude of marker movement and larger PTV (r = 0.137), MLD (r = 0.046), or V20 (r = 0.158) (Figure 4).

## Discussion

Breath coaching and holding using stereotactic body frame has been used as a simple method for SBRT without gating of radiotherapy [6]. However, the effectiveness of visual coaching for tumor localization is still debatable, because of the variations in observers, lengths of observation times, and reproducibility [6,23,24]. Four-dimensional CT with a respiratory gating system using external surrogates has been expected to be useful for respiratory gating. The combination of external surrogates and internal observation with simple prediction models was suggested to reduce the residual motion of the tumor in a simulation study [25]. However, the four-dimensional CT images are all vulnerable to problems relating to the lack of correlation between external surrogates and internal tumor positions during breathing [26-28]. Immobilization in the stereotactic body frame with an abdominal compression device has been used widely [8,9]; however, internal stabilization of tumor location is not certain yet. Planning CT with a slow scan technique can visualize a major part of the trajectory of the tumor by scanning each slice for a time longer than the respiratory cycle [10,11]. With this method, internal target volume can be quite large when the tumor movement is large. Tracking irradiation dynamically pursuing the target with some prediction models has been developed recently bus is still having uncertainty in the modeling of respiratory motion of the tumor [12-17].

The RTRT system potentially resolves these problems described above. With implantation of internal fiducial markers by bronchoscopy, the correlation between internal fiducial markers and internal tumor positions during breathing is more reliable than in the case of external surrogates. Even in the patients with large tumor movement, the target volume margins can be always fixed irrespective of the tumor amplitude. Therefore, target volume does not increase even when the tumor with large movement, which often occurs especially in the lower lobe. Irradiated volume depended only on tumor size using RTRT. Thus, RTRT has potential to reduce the incidence of adverse effect for lung without compromising tumor control comparing other SBRT technology. However, migration of fiducial markers in lung is the potential risk to induce systematic error resulting in higher local relapse rate if we do not take enough care at the daily set-up of the patient [29]. These theoretical advantage and potential disadvantage of SBRT using RTRT system and internal fiducial markers has been debatable.

Insertion of gold markers by bronchoscope is essential for RTRT. Pneumothorax and/or bleeding sometimes occur in trans-bronchial biopsy (TBB) by bronchoscope; however insertion of the gold markers by bronchoscope is much safer than TBB. Pneumothorax and/or bleeding rarely occur in insertion of gold markers by bronchoscope. Imura et al. also reported that markers dropped within the first week after insertion at a high rate and at a much lower rate 1 week after insertion [29]. We usually start RTRT at least 1 week after insertion of the gold markers.

The 3-year LC and OS were 81% and 68%, especially in patients with T1a tumor, the 3-year LC and OS was 92% and 83% in the present study. These were equivalent to several studies of SBRT for Stage I NSCLC shown in Table 4[2,8,10,21,30-34]. However, it is lower than those reported by Timmerman et al., who showed a higher 3-year LC of 97.6% after administering a higher dose of 54 Gy in 3 fractions [8]. The 5-year LC and OS were 78% and 64% in patients with Stage I NSCLC and the 5-year LC and OS was 83% and 75% in patients with T1a tumor in the present study. Asamura et al. and Naruke et al. reported that the 5-year OS was 77.3% and 70.8% for patients with clinical stage IA (T1 tumor) and was 83.9% and 79.0% for patients with pathological stage IA [35,36]. They did not perform FDG-PET at clinical staging, therefore, clinical stage might be underestimated. Our data are compatible with surgical series although phase III comparison is required to prove it.

**Table 4:** Studies of stereotactic body radiotherapy for stage I non-small cell lung cancer

**Author**		Dose* median follow-up		**3yLC**	**3yOS**
**(reference)**	**n**	**(Gy)**	**(mo)**	**(%)**	**(%)**
Onishi (2)	257	30-84(i)	38	NA	57
Timmerman (8)	55	54(p)	34	98	56
Nagata (10)	42	48(i)	30	NA	83 (T1 only)
Onimaru (21)	41	40-48(i)	27	57	47
Uematsu (30)	50	40-60(i)	60	NA	66
Koto (31)	31	45-60(i)	32	78	72
Nyman (32)	45	45(p)	43	80	55
Takeda (33)	38	50(p)	31	93 (T1 only)	90 (T1 only)
Taremi (34)	108	48-60(p)	19	89 (4y)	30 (4y)
Current study	109	48(i) or 40(p)	25	81	68

*Dose was prescribed at the isocenter (i) or periphery (p) of the tumor.

It was very difficult in distinguishing radiation pneumonitis/fibrosis with local recurrence. Local disease recurrence was considered to have occurred only when enlargement of the local tumor continued for > 6 months on follow-up CT scans. FDG-PET and/or histologic confirmation was recommended when local recurrence was suspected. We considered that tumor enlargement only for 3 months might be false positive for local recurrence. In our experience, six months or larger period were thought to be suitable for judgment of the local recurrence. The definition of local control was uncertain in radiotherapy compared with surgical resection.

Matsuo et al. reported that tumor diameter and sex were the most significant prognostic factors in SBRT for NSCLC [11]. In this study, multivariate analysis demonstrated that only the tumor diameter ( ≤ / > 2 cm) was a significant factor in OS. Li et al. reported that FDG-PET/CT was specific in N0 staging for T1–2 NSCLC and the negative predictive value was about 91% in clinical N0 patients, suggested that FDG-PET/CT may help to accurately stage N0 patients and thus identify patients for SBRT [37]. However, there was no statistical significant difference in OS between patients underwent FDG-PET scan before SBRT and those did not in this study.

Grade 3 RP occurred in 2.8% of cases in the present study. The incidence of Grade 3 or greater RP in literature distributed from 0% to 4.9% for patients with stage I NSCLC [2,10,21,31,38]. The severity and incidence of RP in the present study in the present was similar to previous SBRT studies using a similar dose. Timmerman et al. reported a higher incidence of Grade 3 or higher-grade RP of 16.4% with a higher 3-year LC of 97.6% after administering a higher dose of 54 Gy in 3 fractions [8]. The present results indicate that when using SBRT with RTRT the dose to the target may be able to be increased from 48 Gy in 4 fractions to a higher dose such as 54 Gy in 3 fractions and the LC could be improved with a reduction in the incidence of Grade 3 or higher-grade RP. Since the incidence of RP in other SBRT studies is so low, it is not certain yet whether RTRT is any better than other SBRT technology for reducing the incidence of RP by means of a higher dose.

MLD and V20 of the lung volume minus target volume were reported as risk factors of RP [39-43]. Palma et al. also reported that predictors of fatal pneumonitis were daily dose > 2 Gy, V20, and lower lobe tumor location [38]. Comparing non-gated radiotherapy which includes the range of motion adding to CTV to create PTV, the size of PTV is smaller in gated radiotherapy using RTRT in principle irrespective of the tumor amplitude and tumor location. Consequently, MLD and V20 of the lung volume minus PTV should be smaller in the gated radiotherapy using RTRT. Barriger et al. reported that median MLD and V20 were 4.1 Gy and 4% for patients with NSCLC treated by SBRT, respectively [43]. They also reported that median MLD was 5 Gy in patients with RP Grade 2–4, compared with 4 Gy in those with RP Grade 0/1, and median V20 was 6.6% in patients with RP Grade 2–4, compared with 4% in those with RP Grade 0/1, respectively, which were similar to our present study. In the present study, although the maximum amplitude of the marker movement of the lower lobe was significantly greater than that of upper lobe, there was no correlation between larger maximum amplitude of marker movement and larger PTV, MLD, or V20. Present study suggested that SBRT with gated radiotherapy using RTRT system was effective to reduce MLD and V20 and thus the risk of RP consequently.

In this study, we showed the clinical outcomes of SBRT using RTRT for Stage I NSCLC and found that there was no correlation between larger maximum amplitude of marker movement and larger PTV, MLD, or V20. These are risk factors of RP; therefore, RTRT is thought to be useful especially in cases of large amplitude of tumor movement.

## Conclusions

Using only a 5-mm PTV margin to CTV without additional margin for organ motion, SBRT using the RTRT system achieved LC and OS comparable to other SBRT series with very low incidence of RP, which was consistent with the small MLD and V20 irrespective of the tumor amplitude. For stage-I NSCLC, SBRT using RTRT was suggested to be reliable and effective, especially for patients with large amplitude of tumor movement.

## Competing interests

The authors declare that they have no competing interests.

## Authors’ contributions

TI, NK and RO contributed in study design, collection and analysis of data and drafting manuscript. RS performed statistical analysis. SS and KT provided the administrative support. JS-K, NS and SO participated in study design. HS provided the conception of this study and the final approval of the version to be published. And all authors read and approved the final manuscript.
